# Before, Behind and Beyond: Life Stories and Identity in Dementia Care

**DOI:** 10.1111/1467-9566.70254

**Published:** 2026-08-17

**Authors:** Tove Harnett, Glenn Möllergren

**Affiliations:** ^1^ School of Social Work Lund University Lund Sweden

**Keywords:** continuity theory, dementia, identity, life stories, person‐centred care

## Abstract

Life stories are widely promoted in person‐centred dementia care as tools to preserve the identity of the ‘person behind the dementia’. However, as cognitive decline progresses, staff often face dilemmas when current expressions of preference appear to contradict a person's documented past, for example, a devout Muslim requesting pork or a lifelong teetotaller showing interest in alcohol. The aim of this study is to explore how dementia care staff reason about situations in which life story information conflicts with a person’s present identity and expressed preferences. Using Atchley's continuity theory (1989), we explore how staff conceptualise the relationship between external continuity (roles, habits and life patterns) and internal continuity (self‐perception and identity). Based on six focus group interviews with dementia care staff in Sweden, we demonstrate that the frequently cited dichotomy between ‘seeing the person’ and ‘seeing the condition’ is overly simplistic; instead, staff adopt flexible and dynamic understandings of identity that accommodate change over time. Our findings suggest the need to move beyond binary framings and indicate that thinking in terms of the person before, behind and beyond dementia offers a more nuanced framework for understanding identity in dementia care.

## 1 Introduction

Life stories are widely recognised as valuable tools for addressing challenges associated with dementia, a condition described as one that ‘by definition seems to threaten the identity and selfhood of the individual at risk’ (Higgs and Gilleard 2016, 773). The purpose of life stories has been described as preserving residents' unique identities and enabling staff to see the individual behind the condition (Graneheim et al. 2012; McKeown et al. 2010; McKinney 2017; Sweeney et al. 2021). Research on dementia and life stories has for decades focused on cognitivist sensibilities, defining the brain as the legitimate site for action (Fletcher 2023). What makes life stories particularly compelling as an object of study is the strong normative consensus about their importance, which is matched with a lack of clarity about what they are and how they ought to be used in practice.

There is no single universally accepted definition of what constitutes a life story. Dementia UK (2025) describes a life story as ‘a “fact file” about a person living with dementia, encompassing their background, interests and the people and experiences that are important to them’. In Sweden and many other countries, however, there are no standardised procedures for collecting, storing or accessing life stories.

The growing use of life story work in dementia care has been accompanied by an increasing level of academic interest. However, a review of the literature indicates that the majority of studies focus on the significance of life story work as a phenomenon (Grøndahl et al. 2017), whereas comparatively few examine how such narratives are actually used by staff (Clarke et al. 2003; Moos and Björn 2006), their tangible effects (Karlsson et al. 2014) or their relevance for family members (McKeown et al. 2010). Studies have also shown that life stories tend to focus on less problematic aspects of a person's life and often lack information about both earlier trauma and issues related to sexuality and intimacy, aspects that are important in identity construction (Blix et al. 2015; Möllergren and Harnett 2024; Schouten et al. 2023). At the same time, sexuality unfolds as a particularly important aspect in the context of dementia care (Kontos et al. 2016).

Whereas life stories vary greatly in both form and content, most encompass both the external aspects of identity—habits, roles and relationships—and internal aspects such as personality traits and values as they were understood before the onset of dementia. Phrases such as ‘seeing the person behind the patient’ (Clarke et al. 2003) or ‘the person behind the mask of dementia’ (Page 2007) reflect a wider cultural narrative in dementia care that insists on a distinction between the biomedical condition and the lived person. These formulations not only guide everyday practices but also reproduce a sociological framing of dementia that foregrounds identity, dignity and recognition in the face of cognitive decline. Yet, the progression of dementia may challenge the person's previously documented personality and preferences (Graf et al. 2025). An individual known as an early riser might begin to experience morning fatigue; a lifelong vegetarian might develop a preference for meat. Such shifts pose a complex array of ethical and practical challenges for care staff, who must handle the tension between the ‘person behind the condition’ as depicted in their life story and what they perceive as the person's current stated preferences. Similar tensions arise in advance directives and similar documents, when people express their wishes before the onset of dementia as a guiding document, but once they have dementia and express different wishes it places staff in an ethical dilemma about how best to proceed (Bern‐Klug and Levine 2024; Morris et al. 2025). Care staff's involvement in shaping, enacting and relying on the life stories of people with dementia foregrounds the complex bioethical issues of identity, autonomy and representation, issues that have received little sustained attention in the academic literature.

The aim of this study is therefore to explore how dementia care staff reason about situations in which life story information conflicts with a person’s present identity and expressed preferences.

### 1.1 Life Stories and Dementia Care

Sociologically informed perspectives are crucial to personhood and dementia, both for public understanding and for the academic debate (Higgs and Gilleard 2017; Tolhurst et al. 2017). Dementia is generally perceived as a threat to identity, a form of disruption that alters an individual's sense of self and creates a divide between life before and after the onset of the condition (Bury 1991; Atchley 1989). Family members may respond by seeking to preserve the identity of the person ‘behind’ the condition (Fletcher 2021), for instance by creating a life story as a means of maintaining continuity (Gholizadeh et al. 2023). Life stories have been described as tools for self‐continuity and guides for navigating everyday life, serving as a ‘self‐schema’ (Van Woerkum‐Rooker 2025). Similarly, life stories have been promoted as a tool for enabling person‐centred care (Edvardsson et al. 2008), although some studies have called for further research to verify it (Grøndahl et al. 2017).

There are frequent mentions of other benefits of using life stories: the preservation of selfhood and the nurturing of deeper connections between care staff, residents and families (Doran et al. 2019). A review by McKeown et al. (2006) reveals a wide range of aims and approaches to life story work, highlighting its benefits in fostering improved relationships between care staff and family members, as well as providing a deeper understanding of individuals with dementia. The review also raises an ethical concern, however: some older individuals may not wish to have their life stories documented.

Ethical considerations of this kind have generally received less attention in academic explorations of biography‐based approaches to dementia care. Thoresen et al. (2010) highlight the significance of the lifeworld for seriously ill patients and argue that staff should acknowledge the patient's lifeworld—creating an ‘ethical space’ (262) and affirming their existential domain, in order to counter their sense of abandonment and isolation. A related line of reasoning, oriented towards the patient's life history in a broader sense, is developed by Van Nistelrooij et al. (2014). Their discussion is more abstract, offering a philosophical grounding for why biography‐informed care might be ethically important, without engaging directly with biographical methods in practice.

By contrast, Boddington et al. (2024) examine different philosophical accounts of the person in relation to dementia. Drawing on Kitwood (1997) and others, they argue for maintaining personhood, or its multiple manifestations, as a means of mitigating the risk of dehumanising practices in dementia care. This involves recognising the person as they exist with dementia and attending to matters such as ‘the impact of clothing and other aspects of personal grooming’ (104). At the same time, they caution against an exclusive emphasis on the person before dementia. Although their argument implies that a person's life story may be pertinent to sustaining personhood, the paper does not connect this to systematic biography‐based work in dementia care in the Kitwood tradition.

A more determinate strand of bioethical dilemma is found in Sneddon (2020), who analyses advance directives for medical assistance in dying in the context of dementia progression. He asks whether ‘patients who go through deep psychological changes retain their identity’ (706), and whether directives issued prior to dementia should be taken to apply once the person is living with dementia. This is thrown into relief by cases where patients who had earlier stated their intention to end life as dementia developed later appear to enjoy life and experience pleasure when that stage is reached.

Relational perspectives, in contrast to bioethical perspectives, shed light on the value of being recognised according to the situational context, which for example for staff can be both as individuals and as healthcare providers (Sellevoll et al. 2013). Some discussions about person‐centred care raise important questions about such approaches as life story work by critically examining the assumptions about self, identity and person‐centredness (Williamson and Jenkins 2023). Although often framed as enabling recognition and the continuity of the self, they may also rest on a liberal humanist understanding of the person as a coherent, bounded and temporally continuous subject. As Williamson and Jenkins notes, ‘dementia fundamentally challenges us to rethink our understandings of subjectivity, agency, personhood and selfhood in ways that ripple beyond the “human”’ (Williamson and Jenkins 2023, 164). The effort to reconstruct a stable biographical narrative can in that sense be seen as reasserting the very assumptions about the self that dementia disrupts.

Although previous studies differ in scope and theoretical level, together they indicate how bioethical concerns bear directly on people living with dementia, from staff responsibilities to acknowledge the patient's lifeworld, and legislative responses to advance directives, to broader philosophical questions about dehumanisation and the preservation of identity. The supposed benefits of life story work are well documented and include therapeutic reminiscence, enabling a more humane understanding of the person with dementia, providing guidance on preferences and habits in everyday life, and offering insights that help manage anxiety and agitation (Möllergren 2022). These positive outcomes have been observed in various contexts, extending beyond formal care settings to situations such as those involving married couples (Kwak et al. 2018; Miyawaki et al. 2024; Van Hout et al. 2024).

In Sweden, the National Board of Health and Welfare [Socialstyrelsen] (2020) has set a national target that 98% of residents in dementia care homes should have a written life story. To facilitate this practice, care homes commonly provide templates containing preformulated questions that structure the process of documenting residents' lives. These templates typically address personality traits (e.g., whether a person is ‘cheerful, sociable, a morning person, meticulous or short‐tempered’), daily routines, religious beliefs, dietary preferences, lifestyle choices and other biographical details (Möllergren and Harnett 2024). Whereas life story work is often presented as a means of recognising the individuality and personhood of people with dementia, it also raises questions about whose knowledge and interpretations come to shape these narratives. A key challenge concerns the meaningful involvement of people with dementia in producing accounts of their own lives. As cognitive impairment progresses, family members frequently take over the task of writing life stories, potentially shifting the focus away from the voices and perspectives of people with dementia and reducing attention to their ongoing everyday experiences (McKeown et al. 2015).

In recent years, digital life stories have attracted increasing attention due to their potential to support more dynamic and multimodal forms of biographical representation (Dellkvist et al. 2024; Bradley et al. 2023; Elfrink et al. 2018; Dellkvist 2025). Beyond issues of documentation, Rodríguez‐Trejo et al. (2026) suggest that such approaches may also enhance emotional well‐being and social relations by enabling more relational and participatory forms of engagement with personal histories.

As an advocate of a continuity‐oriented professional approach, Kitwood (1997) was among the first to foreground the enduring individual ‘behind’ dementia through personhood‐affirming biographical work. This perspective has subsequently been challenged by more dynamic approaches that conceptualise the identities of people with dementia as continuously unfolding through their changing experiences, relationships and social contexts.

Using a phenomenological approach, Carel (2018, 28) ‘sees illness as a way of living, experiencing the world and interacting with other people’, and argues that illness can be understood as a transformation that not only entails loss, impairment and restrictions but also opens up new horizons and perspectives. Although not specific to the experience of living with dementia, Carel and Kidd (2014) identify an epistemic injustice experienced by people living with medical conditions. Drawing on stigma theory, Young et al. (2019) examines how such epistemic injustice affects people with dementia, demonstrating that stereotypes, prejudice and discrimination can undermine their credibility as knowers and limit their opportunities for meaningful participation in decision‐making and knowledge practices. Professionals are therefore encouraged to recognise the evolving person living with dementia, whose preferences and sense of identity may change over time and differ from, or even conflict with, what is known about the person ‘before’ or ‘behind’ the condition, as documented in a typical life story.

A key gap in the literature concerns how dementia care staff reason about situations in which life story information conflicts with a person's present identity and expressed preferences. Whereass ethnographic work has examined the co‐production of digital life stories (Dellkvist et al. 2026), and interview studies have explored staff perspectives on their use (Melander et al. 2025), less is known about how staff make sense of tensions between biographical accounts and present expressions of identity and preference. Exploring these tensions is important for understanding how care staff conceptualise the relationship between who a person has been, who they are perceived to be in the present and how identity is negotiated in dementia care.

### 1.2 Continuity, Identity and Dementia

To explore aspects of continuity and self, we draw on Atchley's continuity theory (1989) of how individuals maintain a sense of consistency in their lives as they grow older. Although coined for ‘normal’ ageing, we use the theory's core concepts—internal and external continuity—to analyse staff's reasoning about preferences and identity for people with dementia. Internal continuity refers to the maintenance of a consistent sense of self, values and personality traits over time, whereas external continuity relates to the maintenance of continued connections with social roles, places and relationships. We view external continuity as closely intertwined with the maintenance of internal continuity. The preservation of external anchors, such as familiar environments, social roles, relationships and everyday practices, can provide resources through which individuals sustain, express and negotiate a sense of identity and self over time.

There are good grounds for critiquing Atchley's continuity theory for its limited engagement with broader systemic forces and its overly simplified conceptualisation of continuity (Breheny and Griffiths 2017; Rowson and Phillipson 2020). However, we have chosen to employ it as a strictly analytical heuristic rather than an explanatory framework. By using concepts such as internal and external continuity, it becomes possible to examine how care staff articulate and negotiate different versions of identity among people living with dementia.

Life stories typically encompass both internal and external dimensions of continuity, incorporating not only information about an individual's values and personality traits but also external aspects such as food preferences, social roles, familiar environments and habitual practices (Atchley 1989). Whereas Atchley explicitly connects continuity to normal ageing, life story work can be understood as societal or professional support in maintaining continuity in ageing influenced by dementia. In the absence of life story work, such maintenance may also be carried out by family members (Möllergren 2022), who may insist on defending their loved one's right to a certain lifestyle and core values in late‐stage dementia, even if that clashes with what staff may consider the person's wishes.

## 2 Method and Data

This paper is based on a qualitative research design, taking an interpretive approach to explore staff members' experiences of the use of life stories in dementia care. Data were generated from six focus group interviews with staff members in dementia care homes in five Swedish local authorities. Focus groups were chosen as they facilitate not only interaction between participants but also the exploration of shared practices and collective meaning‐making.

We selected the care homes using convenience sampling to ensure variation in geographical location and type of provider (private and publicly operated facilities). The local authorities range in size from 35,000 to 350,000 inhabitants. The names of the dementia care homes have been anonymised (see Table 1).

**TABLE 1:** Care homes, anonymised.

Rosewood gardens	44 beds	Focus group with four nursing assistants
Willowbrook 1	45 beds	Focus group with three nursing assistants
Willowbrook 2	45 beds	Focus group with three nursing assistants
Meadowview	63 beds	Focus group with two nursing assistants, one nurse and one activity leader
Silver pines	59 beds	Interview with two nursing assistants
Oakridge	100 beds	Focus group with three nursing assistants

Five focus group interviews were conducted in person at the care homes and one was conducted over Zoom at the participants' request. The researchers contacted the respective care home managers, who appointed a time and selected a group of staff members to participate. Because staff took part in the focus groups in working hours, most interviews were conducted in the early afternoon when many residents were resting, allowing some staff members to leave the ward. The semi‐structured interview guide included questions about the practical use of life stories in everyday care and how staff become acquainted with residents.

All participants were informed about the purpose of the study and that participation was voluntary. Written informed consent was obtained from all participants before the focus group interviews. There were 17 participants: 15 nursing assistants, one registered nurse and one activity coordinator. The interviews ranged in duration from 49 to 64 min and were transcribed verbatim, resulting in 214 pages of transcript data.

The analysis drew on reflexive thematic analysis as developed by Braun and Clarke (2006, 2019, 2024). In the first phase, we familiarised ourselves with the data through repeated reading of the interview transcripts, developing an overall understanding of the material. In the second phase, we generated initial inductive codes that captured patterns relevant to the research question, such as the different functions of life stories and issues relating to life stories and integrity. We began by coding the material independently before meeting to compare and refine our interpretations.

In the third phase, codes were organised into preliminary themes. At this stage, the analysis was informed by continuity theory, with Atchley’s (1989) concepts of internal and external continuity serving as sensitising concepts. Throughout the analysis, we discussed our interpretations and sometimes disagreed. Our different perspectives were shaped by our professional and disciplinary backgrounds. T.H. has 7 years of experience working in dementia care, including the use of life stories in practice and a background in political science, gerontology and social work. G.M. has a background in journalism and social work. For example, we held different views about what information should be included in life stories and how to balance respect for the privacy of people living with dementia with the potential value of personal information for care staff. Discussing these differences was an important part of our reflexive analytical process and helped us examine our own assumptions.

### 2.1 Limitations

It is in the nature of focus group interviews that participants may align with one another and with what they perceive as the professional imperative or other expected approaches, leaving other representations of individual participants' opinions undisclosed. We did not attempt to verify that what participants said in the focus group interviews corresponded to any observable truth. Similarly, explicitly asking for interviews about life story work risked recruiting a biased selection of care homes or participants, who might be more familiar with the method. Another potential limitation was that the majority of participants were *undersköterskor* or nursing assistants (vocationally trained care staff).

In many countries, including Sweden, nursing assistants work closest to people living with dementia and provide much of the day‐to‐day care. We therefore considered them key respondents in this study. In an international context, Swedish nursing assistant training is comparatively extensive. The programme typically spans 1.5 to 3 years and integrates clinical care with care ethics as an essential component of professional practice. The curriculum emphasises principles such as respect for human dignity, autonomy and integrity in the care of older people, while also addressing confidentiality, respectful communication, culturally sensitive care and the development of professional responsibility in complex care situations (National Board of Health and Welfare [Socialstyrelsen] 2021).

## 3 Results and Discussion: Three Approaches to Identity

We identified three approaches through which staff members discussed the identity of individuals with dementia: (1) the person before dementia, (2) the person behind dementia and (3) the person beyond dementia.

### 3.1 The Person Before

The position of ‘the person before’ centred on relating to the person as they were before the onset of dementia, including their past interests, habits and preferences. Staff described this perspective as particularly emphasised in life stories and often upheld by family members but also increasingly irrelevant. The person they once were was no longer present, and thus their former preferences and routines were often outdated and no longer meaningful to their current care.

And then I’ve noticed that when the family fills this in for their mum or dad, it’s all about how they used to be, before the dementia kicked in, and that’s usually way off from how things are now—what they want, what they like. So yeah, it’s great if you want to know their background, but… yeah.

(Silver Pines, nursing assistant)

The participants expressed frustration that relatives often emphasised information about who the person had been before the onset of dementia. Such accounts may reflect family members' concerns about a perceived loss of continuity in the person's identity. This resonates with the situation described by Bern‐Klug and Levine (2024), where staff in the United States navigate tensions between individuals' current expressions and written advance directives. From the perspective of family members, new preferences and behaviours may be interpreted as symptoms of the condition and as potentially undermining both internal and external dimensions of identity. In this context, life stories can function as a means of maintaining continuity with the person's past self. Care staff, however, tended to interpret changes in preferences and habits as authentic expressions of an evolving identity, expressions they considered important to acknowledge and accommodate in their everyday practice.

Nursing assistant 1 But when they have severe dementia… and they lose all interest.

Nursing assistant 2 …then, then the life story isn't useful anymore, instead you have to…

Nursing assistant 1 Get to know them first.

Nursing assistant 2 …get to know the person…

Nursing assistant 1 A new person, you could say. In the dementia they now have.

(Rosewood Gardens)

At Rosewood Gardens, a dementia care home in southern Sweden, participants in the focus group interview discussed their experiences of getting to know new residents and the part life stories played in that process. To strike a balance between the individual's identity prior to the onset of dementia—as conveyed in their life story—and their preferences and personality expressed in the present, care staff occasionally employed what Wheaton (2022) refers to as ‘therapeutic lies’ to navigate situations that might threaten internal continuity. This required a delicate balancing act between honouring relatives' wishes to preserve the identity of the person as they once were, while respecting the current expressions and needs of the person living with dementia (Long et al. 2024).

One participant described how she had served fruit juice that ‘looked like wine’ to a resident who requested wine during a festive occasion. According to the resident's life story, alcohol consumption was inconsistent with the values and habits that had characterised the resident's earlier life. Serving fruit juice rather than actual wine enabled staff to uphold aspects of the resident's past identity while also responding to the resident's present request, thereby maintaining a degree of internal continuity and supporting the person's sense of self.

Nursing assistant 1 Nah, we don't usually serve that, it's a religious thing, so we got to serve, what's it called… some kind of juice, coloured juice, otherwise it wouldn't be right.

Nursing assistant 2 Yeah, we respect their… what's it called…

Nursing assistant 1 Identity.

Nursing assistant 2 And religion too. (…) Like, we've got a resident on another floor who's Muslim, and she eats halal, so we serve halal. We only serve her halal food. She can't eat, what's it called, you know, the other meat, pork, or whatever. So… (…) You got to respect them too. (…) It's their religion, so…

Researcher But then, maybe it’s just that they don't want pork ’cos they're not used to it, they've never eaten it. But what if one day they suddenly wanted to try it? What would you do then?

Nursing assistant 1 That's tricky. *laughs* (…) Really tricky. I don't know, I mean, we can't force them not to have it. If they want it, we can't say no. Like… yeah, it's tricky. (…)

Nursing assistant 2 Well, what's in their life story, you've kind of got to follow that a bit too. But yeah, I don't know…

Nursing assistant 1 It's important we focus on the person they are now, but we also got to find out what, what they (…) Like, we, we can't just forget who they used to be.

Nursing assistant 2 We got to consider that too. But the most important thing is who they are now.

(Silver Pines, nursing assistants)

Information about both external continuity, such as previous occupations and habits, and internal continuity, such as deeply ingrained beliefs and personality traits, was frequently marginalised on the assumption they were no longer relevant when attending to the person's current needs and preferences:

I mean, we just get a background—what the person used to like, what they used to want to do, hobbies and all that. But honestly, everything changes when they get dementia, from when they were healthy to whatever stage they’re at when they get here.

(Rosewood Gardens, nursing assistant)

This position was particularly evident when participants discussed food preferences and habits. Even though food preferences might not be framed as a core of a person's internal identity, changes could cause friction when family members had strong opinions:

This woman, she wanted to eat cookies, drink juice. She didn’t want to listen to church music, and she really didn’t want to do gymnastics. But we had to keep trying to make her do it anyway [despite the daughter’s insistence], and in the end, we staff were just like, ‘No, we’re not doing this anymore—we’re going to meet your mum where she’s at.’ And yeah, that caused quite a bit of trouble. But, yeah, that’s the thing—it can be a problem, ’cos when it comes to dementia care the family is often really involved in everything, every decision.

(Silver Pines, nursing assistant)

In that particular case, the woman's adult children had written in her life story that she enjoyed listening to church music and participating in group exercise sessions, which can be understood as an attempt to maintain a sense of external continuity and a stable narrative of personhood over time. However, the care staff did not support these descriptions, which, as they reported, led to conflict with the family. This tension reflects what Melander et al. (2025) describe as potential discrepancies between life story documentation and staff members' situated real‐time observations in everyday care practice.

Yet, at the same time, the care staff often saw biographical information—what some referred to as ‘just getting a background’—as highly valuable, especially when managing episodes of unrest. Referring to early life memories was consistently highlighted as one of the key benefits of life story work, providing staff with tools to offer comfort and stability at challenging moments.

### 3.2 The Person Behind

The second position, ‘the person behind’, involved staff interpreting certain behaviours as expressions of ‘the dementia speaking’, while maintaining a belief that there remained a person behind the behaviours. This differed from the notion of ‘the person before’, which concentrated on continuity expressed in external markers such as interests, relationships and routines. Instead, this position reflected a focus on internal continuity, attending to deeper dimensions of personality, values or moral character. For example, when residents exhibited physically aggressive behaviour, staff sometimes interpreted this not as a reflection of the person's true self but as a symptom of their medical condition, thereby preserving a sense of the individual's core identity behind the surface.

There, it’s like, no problem to let go of who the person used to be. But if someone gets physically aggressive and violent because of their illness, then you have to see the person behind the illness, ’cos that’s what really matters. The violence isn’t them, you know. (…) But when it’s the other way around, and it’s nothing serious—like, what does it matter if your mum drinks juice? It doesn’t. But if your mum starts hitting people—yeah, sure, that’s a problem. But it’s still not her, not really. (…) You’ve got to be able to tell the difference.

(Silver Pines, nursing assistant)

With statements such as ‘it's not really her’ and ‘you have to see the person behind the condition’, staff acknowledged that individuals with dementia have an identity somehow hidden by the condition. It also implies that certain behaviours can be framed as expressions of dementia, and so not attribute them to the person's ‘true’ identity. This echoes Storm and Lowndes's findings (2021) that staff excused racist behaviour among care home residents by referring to their medical conditions.

Participants highlighted another dimension of the ‘person behind’: information about challenging life experiences was often absent from life story documents. This may partly reflect the limited scope of life story templates, which rarely include questions addressing such experiences (Möllergren and Harnett 2024). However, family members may also be unaware of certain aspects of their relatives' lives, or the sensitive and potentially taboo nature of particular experiences may discourage their disclosure and documentation (Simonhjell and Hellstrand 2019). From the staff members' perspective, accessing such information was often an ongoing process, involving the gradual piecing together of fragments of a person's life history over time. During episodes of distress, specific words or behaviours could provide clues to past experiences that might help explain the person's reactions. On occasion, family members could contribute details that helped paint a fuller picture. When such knowledge became available, it was sometimes framed as a way of understanding the ‘person behind’:

Yeah, they’ve been through different times, and maybe they had a period that was really rough or something, you know. (…) I’ve actually got this resident; she’s told me a lot about when she was little (…). And her mum, actually, like, used to beat her just ’cos she was left‐handed. (…) It was a shameful thing back then, you know. So, like, sometimes they get depressed, maybe some old trauma comes up… (…) Yeah, it is trauma, that’s what it is. So, it’s no surprise some of them end up depressed, ’cos stuff like that, you never forget.

(Rosewood Gardens, nursing assistant)

Knowing the woman had been traumatised by her mother telling her she would never be able to manage a household because she was left‐handed, the staff could frame certain behaviours as expressions of the person behind the dementia. At another dementia care home, the staff described a woman whose behaviour they initially did not understand, but once they were aware of her past trauma they had a better understanding of the person behind the condition and why she was so afraid of fire.

There was a woman once, her house had burned down, and she was inside. She was, well, yes, they saved her. One time she was sitting and watching TV, and there was something about a fire on TV, and she screamed. And I have to be honest, we missed it. We went back and read her life story, and it said that there had been a fire, and it stayed with her. Then I thought, yes, it was an event, so maybe it’s not a good idea to just sit in front of the TV and watch programmes about fires.

(Willowbrook 2, nursing assistant)

Other traumatic events included war, violence and conflict. One staff member described a previous resident who had survived the Holocaust. This information meant the staff saw the person behind the condition and framed her fear of showers in the light of it:

Nursing assistant 1: I mean, it can really set something off too, like if they see something on TV. I know there used to be a lot of talk about this, like, with Jewish people who’d been gassed—how just going into the shower could bring back massive trauma. And that’s why it’s so important to know—okay, this person was in a concentration camp, they went through something horrific. You can’t just be like, ‘Alright, time for a shower!’ You got to think about it, work around it. How do we do this in the best way so we’re not dragging up awful memories?’

Nursing assistant 2: Yeah, talking about showers, I had this one woman—every time we tried to shower her, she’d panic. We had no idea why, and then she just goes, ‘You’re trying to kill me…’.

(Meadowview, nursing assistants)

This was typical of the staff's approach to recognising the person behind the dementia diagnosis as someone defined not merely by previous preferences and habits but by experiences that must be taken into account in order to provide quality care.

### 3.3 The Person Beyond

The third position identified was the ‘person beyond’. This perspective shifts attention towards how people with dementia continue to express personality and preferences in the present, while living with the condition. It involved staff engaging with individuals' current expressions and recognising these as meaningful aspects of identity. Importantly, it did not imply reducing the person to their diagnosis but rather reflected an understanding that present expressions, even when shaped by dementia, remain part of the person's evolving identity.

Some dementia care guidelines frame the situation as a person–dementia dyad: staff must focus either on the person or on the condition. In contrast, the ‘person beyond’ perspective illustrates a more nuanced approach, where staff relate to who the person is now, while acknowledging the impact of dementia. Within this framework, participants discussed the continuity of internal identity structures from multiple angles. This perspective bears similarities to Sellevold et al.’s (2013) notion of how to accept the patient with dementia as an individual. One example in this study was that of a lifelong teetotaller with advancing dementia, who began expressing a desire to drink alcohol. In that case, staff eventually chose to serve him alcohol, acknowledging the person's present preferences to be a valid expression of identity, even when it appeared to contradict his personal values.

We had a liqueur tasting and there was this new guy who’d just moved in. His wife came to visit, and they were sitting there, having a great time—she was so happy about the nice moment they were having. But then she asks, ‘What is it they’re tasting?’ So, we tell her ‘It’s liqueur.’ And man, her whole world just fell apart. (…) ’Cos he’d never touched a drop of alcohol in his life, and now here we were, giving him liqueur. But he loved it—thought it was great, really enjoyed himself. Yeah… but then we had to deal with the wife. Had to talk to her, calm her down. In the end, he still got to taste the liqueur, but she never wanted to see that happen again. (…) It was all for her really—they’d been totally against alcohol their whole lives, even their parents before them.

(Meadowview, nursing assistant)

The man's wife had become visibly distressed when she witnessed what she saw as a break with the person her husband used to be—specifically, a departure from his internal continuity. Whereas she was focused on preserving the identity he had before, the staff related more to the person he was beyond dementia. As one staff member put it, ‘he loved it—thought it was great, really enjoyed himself’, a response that validated their decision to honour his current wishes. The man had been a teetotaller, but the person beyond dementia—the individual as he was now—clearly wanted to drink. In contrast to Sellevold et al.’s concept of understanding the patient's expression (2013), the situation above can be interpreted in terms of upholding external continuity by being in a social setting and accepted as a person with self‐determination.

Meals in particular are often described as being central to maintaining external structures of identity, and life story documents can have detailed information about food preferences (Möllergren and Harnett 2024). Participants in the present study noted that tensions could arise when individuals developed new preferences that clashed with deeply rooted aspects of their identity, especially when those preferences conflicted with ideological or religious values. The statement ‘most important is the person that exists now’ emphasises a position to see the ‘person beyond’, with the ‘person before’ perceived as withering away. Although external continuity may involve the maintenance of observable behavioural patterns—such as abstaining from meat—the presence of internal continuity can only be meaningfully validated by referring to the individual's personal beliefs about what is typical for them, such as identifying as a committed vegetarian (Atchley 1989).

Participants said the continuity of some habits and preferences was more important than others for maintaining a person's identity. However, there was little clarity about the habits and preferences that were to be preserved at all costs. In some cases, maintaining continuity based on religious affiliation was considered important, but in others, not:

We had a Muslim woman. When she moved in, we were informed that she was Muslim, so she shouldn’t eat pork and ideally should not drink alcoholic beverages. She was from Bosnia. So, we respected that, but after some time, she got worse, meaning her condition, dementia, deteriorated, and she wanted to eat what the others were eating. She no longer wanted what she had on her plate, even though it had the same potatoes, sauce, and vegetables as the others, but the meat was different. She was very determined that, ‘I want to eat like they do,’ and became almost angry, actually. So, we served her as she wanted. And we turned to the family members: ‘This and this has happened, and mom has changed, and now she wants to eat exactly like everyone else.’ They said, ‘Mum should have what she wants.’ They were wholly respectful, and then after a while she wanted beer, so we gave it to her, actually, because she had forgotten that she was Muslim, and she sat together with others, and she wanted exactly what everyone else had, and she got it.

(Meadowview, nursing assistant)

Serving pork and beer to a Muslim could be a violation of the internal continuity of the person's identity; however, rather than frame it like that, staff saw it as a question of autonomy and equal treatment. The woman said she wanted to choose what to eat and that meant the same food as others at the table. Meeting her wishes thus became a matter of avoiding negative discrimination rather than a violation of her identity. Rather than treating identity as a fixed, internally coherent essence, the situation illustrates how preferences are negotiated in a social context. The wish to belong—in this case, to share a meal with neighbours—could itself be understood as an expression of internal continuity, reflecting enduring needs for connection, participation and social belonging, even when it diverges from aspects of the person's biographical past.

This observation resonates with Jenkins's critique (Williamson and Jenkins 2023) of person‐centred care, which suggests exactly that: that dementia unsettles such individualised understandings of the self. Yet, the critique does not render biographical approaches altogether irrelevant; rather, it highlights a tension within practice. In our empirical material, staff do not simply recover pre‐existing life stories, but engage in ongoing interpretive work, sometimes piecing together fragments from different situations in order to make sense of expressions of changed preferences. This process does not so much restore the bounded individual as it produces a situationally workable account that can guide care.

In that sense, life story work is less the preservation of an intact personhood, and more a practice by which staff prioritise certain aspects of the person's expressions over others. The woman's present wish to share the meal with others was given precedence over biographical or culturally anchored understandings of her identity. Acknowledging the critique of person‐centred care by Williamson and Jenkins (2023) thus allows for a more precise analytical framing: rather than treating identity as something stable to be upheld, the example highlights how care practices may actively prioritise social belonging in a given situation, even when it entails setting aside elements of the person's life story.

## 4 Conclusions

This study contributes to ongoing debates about how identity and personhood are understood in the context of dementia (Boddington et al. 2024; Van Woerkum‐Rooker 2025), as well as to discussions of human rights and ethical approaches in dementia care (Kontos 2005; Kontos et al. 2016). Recent work has moved towards more situation‐specific analyses of the relationship between the ‘then’ and ‘now’ selves of people with dementia, as well as staff practices in negotiating those selves. There have also been important theoretical contributions about personhood, dementia and change over time (Tewes 2021). Our ambition has been to add to this body of research by examining how such issues are approached in everyday care practice.

Our findings shed light on staff members' perceptions of the usefulness of life stories, particularly for how they navigate the tensions between a person's current preferences and those expressed before the onset of dementia. In Atchley's framework (1989), internal and external continuity are interwoven, with external continuity often supporting internal continuity. External stability may function as a mirror that reflects and sustains the inner self, for example by enabling continuity in familiar routines, foods or activities. A life story could be seen as a resource that supports both forms of continuity. However, our findings suggest that this interconnectedness is not strongly reflected in how staff conceptualise identity in practice.

Staff generally described life story information as useful, especially in relation to a person's background and difficult experiences; however, they put less emphasis on personality traits, interests or everyday preferences, and did not typically view life stories as tools for maintaining external continuity. With regard to internal continuity, biographical information alone did not appear to be thought a means of sustaining a stable personal identity. Rather, staff primarily used life stories in situational ways, for example to de‐escalate distress and provide reassurance in moments of confusion or disorientation.

This practice raises important questions about individual integrity and narrative authority. When a person cannot consent to the reconstruction of their life history, particularly traumatic experiences, the ethical grounds for doing so become uncertain. Yet, withholding information that may reduce distress is equally problematic. The question of whose story comes to shape care therefore remains unresolved. As life stories are continuously constructed in practice, they may increasingly reflect staff interpretations rather than the person's own perspective, highlighting tensions around narrative identity and indeterminacy (Van Nistelrooij et al. 2014; Sneddon 2020).

Our findings also align with narrative approaches to memory that challenge the notion of memory as a stable repository of experience. Rather than being retrieved intact, memories are constructed in the present, often through fragmented and non‐linear processes (Brockmeier 2015). Staff members' efforts to piece together fragments can thus be understood as ongoing acts of narrative reconstruction through which past experiences and present expressions are jointly made meaningful.

Taken together, this study complicates the assumption that person‐centred care should primarily be oriented towards uncovering the person ‘behind’ dementia. At the same time, our findings demonstrate that life histories and biographical information can remain valuable resources for providing context‐sensitive care. In dialogue with research on identity and selfhood, including Kontos's (2005) notion of embodied selfhood, we suggest that an exclusive focus on a prior or ‘hidden’ identity risks overlooking how selfhood continues to be expressed through embodied, relational and situated experiences. Rather than reinforcing a distinction between person and disease, our findings highlight the limitations of such binary understandings of identity in dementia care. We therefore propose a more nuanced framework in which the person before, behind and beyond dementia are understood as interconnected perspectives that together capture the ongoing and evolving nature of identity. Such an approach provides a more comprehensive basis for understanding personhood in dementia and for informing care practices.

## Author Contributions

**Tove Harnett:** conceptualization, investigation, funding acquisition, writing – original draft, writing – review and editing, validation, methodology, formal analysis, supervision, resources, project administration. **Glenn Mollergren:** conceptualization, investigation, writing – original draft, writing – review and editing, validation, methodology, formal analysis.

## Funding

This work was supported by the Ribbingska Memorial Foundation through funding for the project *Life Stories in Elder Care: Challenges and Opportunities*.

## Ethics Statement

This study was performed in accordance with the Declaration of Helsinki. According to the Swedish Ethical Review Authority, this study does not require ethical review, as it does not involve the processing of sensitive personal data. Therefore, the research design has been discussed and approved by a local ethics committee at the authors’ university.

## Consent

All participants were fully informed about the purpose of the study, and participation was entirely voluntary. Written informed consent was obtained from all participants prior to their involvement in the focus group interviews.

## Conflicts of Interest

The authors declare no conflicts of interest.

## Data Availability

Because of ethical concerns, supporting data cannot be made openly available. Further information about the data and conditions for access is available at the Lund University data archive: dataskyddsombud@lu.se.
